# Supplementary figures and images for: Simple predictive models identify patients with COVID-19 pneumonia and poor prognosis
Source: PLoS One. 2020 Dec 28;15(12):e0244627. doi: 10.1371/journal.pone.0244627 (PMC7769554; doi:10.1371/journal.pone.0244627)

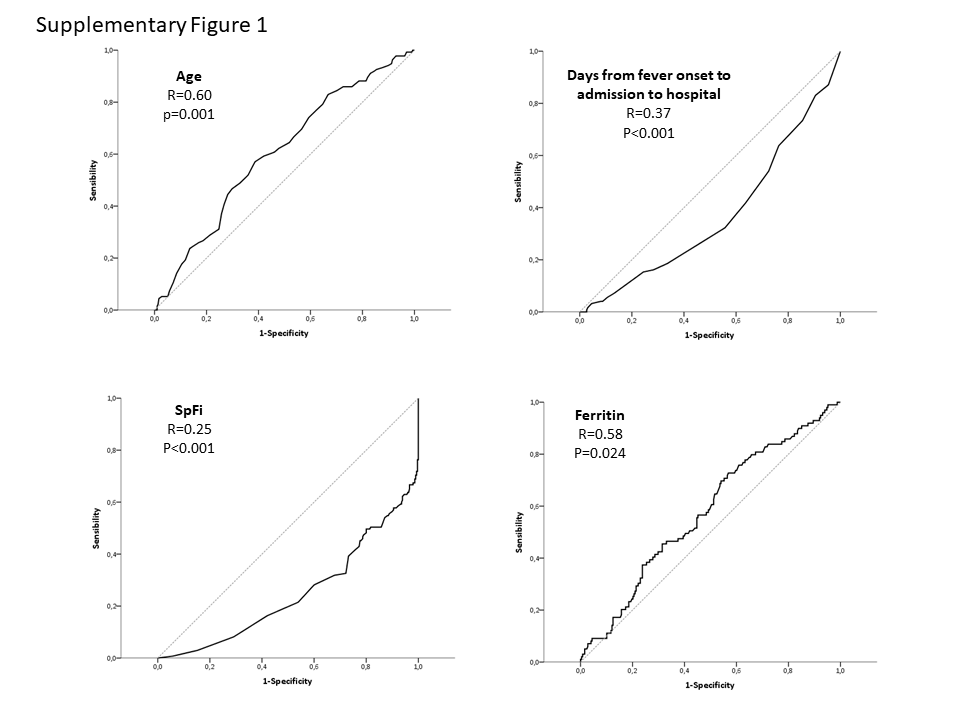

Supplement: S1 Fig — (TIF) [file pone.0244627.s001.tif]

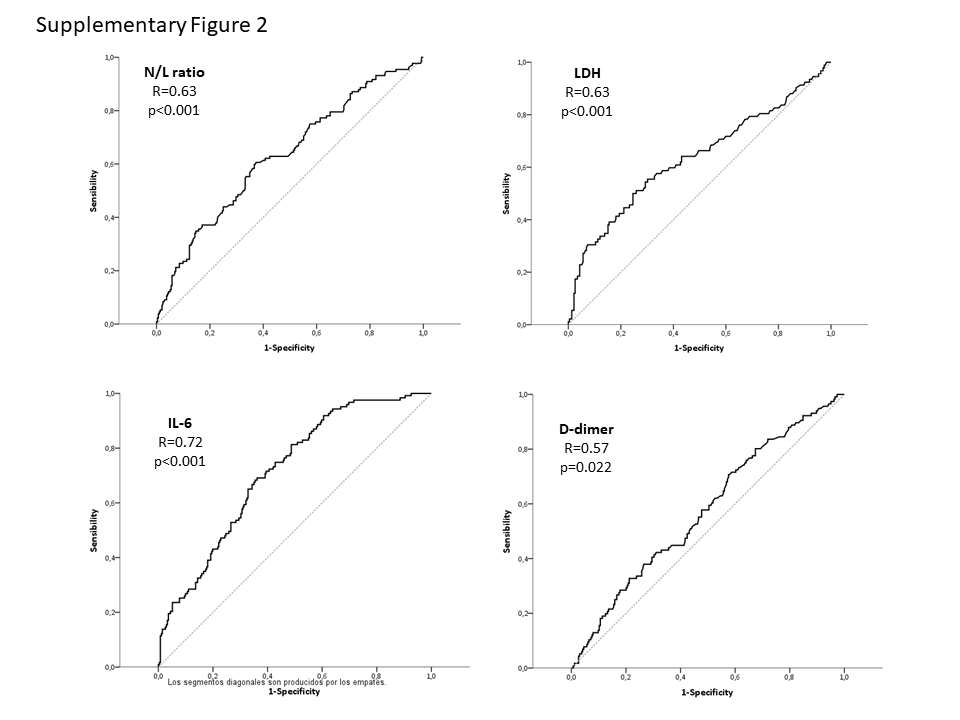

Supplement: S2 Fig — (TIF) [file pone.0244627.s002.tif]
