# Supplementary material for: Simple predictive models identify patients with COVID-19 pneumonia and poor prognosis
Source: PLoS One. 2020 Dec 28;15(12):e0244627. doi: 10.1371/journal.pone.0244627 (PMC7769554; doi:10.1371/journal.pone.0244627)
Supplement: S1 Table — (PDF) [file pone.0244627.s003.pdf]

S1 Table. Treatment and clinical endpoints according to the high-flow oxygen support (invasive or non-invasive ventilation, nasal high-flow cannula or non-rebreathing oxygen mask with reservoir).

	**Overall (N=430)**	**High-flow oxygen support**		**p value**
		**Yes (N=135)**	**No (N=295)**	
**Antiviral treatment**				
None	38 (8.8%)	11 (8.1%)	27 (9.2%)	0.961
Lopinavir/R (LPV/R)	3 (0.7%)	1 (0.7%)	2 (0.7%)	
Hidroxycloroquine (HCQ)	96 (22.3%)	32 (23.7%)	64 (21.7%)	
LPV/R + HCQ	293 (68.1%)	91 (67.4%)	202 (68.5%)	
**Tocilizumab**	120 (27.9%)	84 (62.2%)	36 (12.2%)	<0.001
**Pulses of corticoids***	53 (12.3%)	38 (28.1%)	15 (5.1%)	<0.001
**Days from admission to high-flow oxygen support**	2.3±3.2	3.4±3.4	---	---
**Days of hospitalization**	12.2±10.4	25.3±13.2	8.0±3.8	<0.001
**Overall mortality rate**	34 (7.9%)	30 (22.2%)	4 (1.4%)	<0.001
**Mortality rate due to covid-19**	24 (5.6%)	21 (15.6%)	3 (1%)	<0.001
**Readmision related to covid-19 complications**	15 (3.5%)	3 (3.1%)	12 (4.3%)	0.428

*Defined as ≥ 1 mg/Kg/d for at least 3 days.
