# Supplementary material for: Simple predictive models identify patients with COVID-19 pneumonia and poor prognosis
Source: PLoS One. 2020 Dec 28;15(12):e0244627. doi: 10.1371/journal.pone.0244627 (PMC7769554; doi:10.1371/journal.pone.0244627)
Supplement: S2 Table — (PDF) [file pone.0244627.s004.pdf]

S2 Table. Univariate and multivariate analysis of factors associated with mortality rate related to SARS CoV-2

	Univariate analysis	Multivariate analysis	
	**p value**	**95% CI**	**p value**
**Male sex**	0.022	--	0.190
**Age, years**	<0.001	--	0.164
**Arterial Hypertension**	<0.001	4.485 (1.725-11.661)	0.002
**Diabetes mellitus**	0.076	--	0.533
**Obesity**	0.430	--	--
**Chronic obstructive pulmonary disease**	0.001	--	0.776
**Asthma**	0.265	--	--
**Smoker (active or former)**	<0.001	--	0.088
**Immunosuppresive therapy**	0.607	--	--
**X-ray (**peripheral infiltrate as reference**)**<br>Unilateral pneumonia<br>Bilateral pneumonia	0.064	--	0.539
**SpO2/FiO2**	<0.001	0.996 (0.993-0.999)	0.012
**Fever**	0.200	--	--
**Cough**	0.022	--	0.441
**Dyspnea**	0.403	--	--
**Diarrhea**	0.026	--	0.271
**Myalgia**	0.051	--	0.278
**Hemoglobin, mg/dL**	0.011	--	0.173
**Neutrophils/ Lymphocyte ratio**	0.042	--	0.325
**Platelets, x10E9/L**	0.105	--	--
**Ferritin, ng/mL**	0.208	--	--
**C reactive protein, mg/dL**	0.003	--	0.985
**LDH, IU/mL**	0.196	--	--
**ALT, IU/mL**	0.076	--	0.132
**Conjugated bilirubin, mg/dL**	0.071	--	0.755
**Urea, mg/dL**	<0.001	--	0.364
**IL-6, pg/mL**	0.792	--	--
**Antiviral therapy (**none as reference**)**<br>HCQ<br>HCQ + LPV/R	0.077	--	0.153
