# Supplementary material for: Simple predictive models identify patients with COVID-19 pneumonia and poor prognosis
Source: PLoS One. 2020 Dec 28;15(12):e0244627. doi: 10.1371/journal.pone.0244627 (PMC7769554; doi:10.1371/journal.pone.0244627)
Supplement: S3 Table — (PDF) [file pone.0244627.s005.pdf]

S3 Table: Number of patients with need of oxygen supplementation at admission to the ER department and the mean SpO2/FiO2 according to the baseline chest X-ray findings.

		**Chest X-ray (N=430)**			**p value**
		**Peripheral infiltrate (N=198)**	**Unilateral pneumonia (N=77)**	**Bilateral pneumonia (N=232)**	
**Baseline oxygen support to achieve SpO2>95%**	**None (0.21)**	152 (76.8%)	62 (80.5%)	83 (53.5%)	<0.001
	**0.24-0.34**	36 (18.2%)	13 (16.9%)	30 (19.4%)	
	**≥0.35**	10 (5.1%)	2 (2.6%)	42 (27.1%)	
**Mean SpO2/FiO2 (SpFi)**		424±75	438±54	347±139	<0.001
